# Curcumin Significantly Enhances Dual PI3K/Akt and mTOR Inhibitor NVP-BEZ235-Induced Apoptosis in Human Renal Carcinoma Caki Cells through Down-Regulation of p53-Dependent Bcl-2 Expression and Inhibition of Mcl-1 Protein Stability

**DOI:** 10.1371/journal.pone.0095588

**Published:** 2014-04-17

**Authors:** Bo Ram Seo, Kyoung-jin Min, Il Je Cho, Sang Chan Kim, Taeg Kyu Kwon

**Affiliations:** 1 Department of Immunology, School of Medicine, Keimyung University, Daegu, South Korea; 2 College of Oriental Medicine, Daegu Haany University, Gyeongsan, Korea; 3 Medical Research Center for Globalization of Herbal Formulation, Daegu Haany University, Gyeongsan, Korea; University of Kansas Medical Center, United States of America

## Abstract

The PI3K/Akt and mTOR signaling pathways are important for cell survival and growth, and they are highly activated in cancer cells compared with normal cells. Therefore, these signaling pathways are targets for inducing cancer cell death. The dual PI3K/Akt and mTOR inhibitor NVP-BEZ235 completely inhibited both signaling pathways. However, NVP-BEZ235 had no effect on cell death in human renal carcinoma Caki cells. We tested whether combined treatment with natural compounds and NVP-BEZ235 could induce cell death. Among several chemopreventive agents, curcumin, a natural biologically active compound that is extracted from the rhizomes of Curcuma species, markedly induced apoptosis in NVP-BEZ235-treated cells. Co-treatment with curcumin and NVP-BEZ235 led to the down-regulation of Mcl-1 protein expression but not mRNA expression. Ectopic expression of Mcl-1 completely inhibited curcumin plus NVP-NEZ235-induced apoptosis. Furthermore, the down-regulation of Bcl-2 was involved in curcumin plus NVP-BEZ235-induced apoptosis. Curcumin or NVP-BEZ235 alone did not change Bcl-2 mRNA or protein expression, but co-treatment reduced Bcl-2 mRNA and protein expression. Combined treatment with NVP-BEZ235 and curcumin reduced Bcl-2 expression in wild-type p53 HCT116 human colon carcinoma cells but not p53-null HCT116 cells. Moreover, Bcl-2 expression was completely reversed by treatment with pifithrin-α, a p53-specific inhibitor. Ectopic expression of Bcl-2 also inhibited apoptosis in NVP-BE235 plus curcumin-treated cells. In contrast, NVP-BEZ235 combined with curcumin did not have a synergistic effect on normal human skin fibroblasts and normal human mesangial cells. Taken together, combined treatment with NVP-BEZ235 and curcumin induces apoptosis through p53-dependent Bcl-2 mRNA down-regulation at the transcriptional level and Mcl-1 protein down-regulation at the post-transcriptional level.

## Introduction

The phosphoinositide 3-kinase (PI3K)/Akt and mammalian target of rapamycin (mTOR) signaling pathway is important for many cellular functions such as cell proliferation, growth control, metabolism, and cell survival. In cancer, PI3K-Akt-mTOR is activated via multiple mechanisms, including phosphatase and tensin homolog (PTEN) mutation (PI3K-Akt signaling negative regulator) [1], [2], Akt overexpression [3], [4], and the activation of upstream signaling pathways (receptor tyrosine kinase and Ras) [5], [6] that are associated with cancer cell proliferation, tumor growth, metastasis, and cell survival [7]–[10].

mTOR is composed of two functionally different multiprotein complexes, TORC1 and TORC2. TORC1 is composed of mTOR, mammalian LST8 (mLST8), proline-rich Akt substrate 40 (PRAS40), and raptor (regulatory-associated protein of mTOR), while TORC2 contains mTOR, mLST8 (GβL), mSIN1, PRR5 (protor), and rictor (rapamycin-insensitive companion of TOR) [11]–[14]. TORC1 is rapamycin-sensitive; thus, rapamycin induces the de-phosphorylation of TORC1 substrates [eukaryotic initiation factor 4E-binding protein 1 (4E-BP) and S6 kinase 1 (S6K1)] [15]. In contrast, TORC2 is known as a rapamycin-insensitive complex, and it modulates Akt phosphorylation at serine 472 [15]. TORC1 inhibitors, such as temsirolimus and everolimus, are used to treat patients with renal cell carcinoma, but only a small population of patients have good responses to these drugs [16], [17]. Furthermore, only TORC1 inhibition can activate TORC2 signaling, resulting in the activation of Akt [18]. Therefore, inhibition of TORC1/2 could improve therapeutic efficiency.

Since PI3K/Akt/mTOR signaling is hyperactivated in renal cell carcinoma (RCC), inhibition of PI3K/Akt/mTOR pathway is one of target for cancer treatment [19]–[21]. Although inhibitors of PI3K/Akt have anti-cancer effect in pre-clinical studies [19], however, the clinical use of inhibitors (LY294002 and wortmannin) is limited due to several problems. For examples, both inhibitors did not have specificity against PI3K family members, low solubility and aqueous instability [22], [23]. mTORC1 inhibitors (temsirolimus and everolimus) have approved for the treatment of patient with RCC. However, many patients have acquired drug resistance during treatment, due to feedback activation of PI3K/Akt [24]. Therefore dual PI3K/Akt/mTOR inhibitor is more effective to treatment against RCC.

NVP-BEZ235 is a PI3K/Akt and mTOR inhibitor. NVP-BEZ235 inhibits class 1 PI3K activity via binding to its ATP-binding domain, and it also obstructs TORC1 and TORC2 activity via binding to their ATP-binding domain [25]. NVP-BEZ235 has a cytotoxic effect on T-cell acute lymphoblastic leukemia [26] and Waldenstrom macroglobulinemia [27], and it has a growth inhibitory effect in hepatocellular carcinoma cells [28] and ovarian cancer cells [28]. In RCC, NVP-BEZ235 also has anti-cancer effects. NVP-BEZ235 reduced viability and cell proliferation [21], [29], [30]. Although NVP-BEZ235 is a more effective strategy to enhance cancer treatment than the inhibition of only TORC1 or PI3K/Akt, the effect of NVP-BEZ235 on apoptosis in renal carcinoma cells is not well characterized. Furthermore, since NVP-BEZ235 is reversible inhibitor, inhibition effect of PI3K/Akt/mTOR is transient [25]. Therefore, to overcome the drug resistance and improve clinical effects, evaluation of novel therapeutic strategy that have maintain anti-cancer effect and less toxicity for normal cell are important.

Curcumin, which is a polyphenolic phytochemical extracted from the rhizomes of the *Curcuma longa* plant, has multiple functions including anti-tumor, anti-inflammatory, and immune modulatory effects [31]–[33]. In particular, curcumin induces cell death in several types of cancer cells. For example, in our previous study, curcumin (>50 µM) induced apoptosis through the production of reactive oxygen species (ROS), and the down-regulation of Bcl-xL and inhibitor of apoptosis protein (IAP) in Caki cells [34]. In addition, curcumin also increased apoptosis in B-cell lymphoma [35], colon carcinoma [36], gastric carcinoma [37], Ehrlich's ascites carcinoma cells [38], melanoma [39], and multiple myeloma [40]. Furthermore, curcumin has a synergistic effect with other anti-cancer drugs. Our group and others reported that curcumin sensitized tumor necrosis factor-related apoptosis-inducing ligand (TRAIL)-induced apoptosis [41]–[43], increased radio sensitivity [44], [45], and potentiated the anti-cancer effect of 5-fluorouracil and gemcitabine [46], [47].

We consider that the combination therapy of molecularly targeted anticancer agents provide new approaches to improve the effectiveness of therapy for cancer. Many researchers investigate mechanisms and effects of combination therapy to induce cell death in cancer cells. In this study, we investigated whether natural compounds enhance NVP-BEZ235-induced PI3K-Akt-mTOR signaling inhibition and cell death in human renal carcinoma Caki cells, and the molecular mechanisms underlying co-treatment with curcumin and NVP-BEZ235 were analyzed in human renal carcinoma Caki cells.

## Materials and Methods

### Cells and Materials

Caki, ACHN, A498, MDA-MB231 and U87MG cells were purchased from the American Type Culture Collection (Manassas, VA). The normal human skin fibroblasts (HSFs) cells were purchased form Korea Cell Line Bank (Seoul, Korea). Primary cultures of human mesangial cells (MCs) were purchased from Clonetics (San Diego, CA). The cells were cultured in Dulbecco's modified Eagle's medium that contained 10% fetal bovine serum, 20 mM HEPES buffer, and 100 mg/ml gentamicin. The PCR primers were purchased from Microgen (Seoul, Korea). NVP-BEZ235 was purchased from LC Laboratories (Woburn, MA). Curcumin was purchased from Biomol (Biomol Research Laboratories, Inc., PA). Kahweol was purchased from LKT Labs (St. Paul, MN). Anti-pro-caspase 3, anti-cleaved caspase 3, anti-DR4, anti-phospho S6K, anti-phospho-Akt, anti-PSMD/S5a, and anti-PSMA5 antibodies were purchased from Cell Signaling Technology (Beverly, MA). Anti-DR5, anti-Bcl2, anti-Bcl-xL, anti-Mcl-1, anti-XIAP, anti-cIAP2, anti-cFLIP, anti-p53, and anti-PARP antibodies were purchased from Santa Cruz Biotechnology (Santa Cruz, CA). The anti-actin antibody was obtained from Sigma (St. Louis, MO). Lactacystin was purchased from ENZO (Enzo Biochem Inc., NY). Pifithrin-α was obtained from Millipore (Bedford, MA). Triptolide was purchased from Alexis Biochemical (San Diego, CA). z-VAD-fmk (a pan-caspase inhibitor) and baicalein were obtained from Calbiochem (San Diego, CA), and the other chemicals were purchased from Sigma (St. Louis, MO).

### Western Blot Analysis

Cells were washed with cold PBS and lysed on ice in modified RIPA buffer (50 mM Tris–HCl, pH 7.4, 1% NP-40, 0.25% Na-deoxycholate, 150 mM NaCl, 1 mM Na_3_VO_4_, and 1 mM NaF) containing protease inhibitors (100 µM phenylmethylsulfonyl fluoride, 10 µg/ml leupeptin, 10 µg/ml pepstatin, and 2 mM EDTA). Lysates were centrifuged at 13,000×g for 15 min at 4°C, and the supernatant fractions were collected. Proteins were separated by SDS-PAGE and transferred to an Immobilon-P membrane. Specific proteins were detected using enhanced chemiluminescence.

### Flow Cytometry Analysis

Approximately 1×10^6^ cells were suspended in 100 µl PBS, and 200 µl 95% ethanol was added while vortexing. The cells were incubated at 4°C for 1 h, washed with PBS, and resuspended in 250 µl 1.12% sodium citrate buffer (pH 8.4) together with 12.5 µg RNase. Incubation was carried out at 37°C for 30 min. The cellular DNA was then stained by applying 250 µl propidium iodide (50 µg/ml) for 30 min at room temperature. The stained cells were analyzed by fluorescence activated cell sorting (FACS) on a FACScan flow cytometer (E5464, Becton Dickinson, USA) for relative DNA content based on red fluorescence.

### Cell Viability Assay

The cytotoxic effect was investigated using a commercially available proliferation kit (WelCount Cell Viability Assay Kit, WELGENE, Daegu, Korea). Briefly, the cells were plated in 96-well culture plates at a density of 3,000 cells/well in phenol red free medium and allowed to attach for overnight. After treatment with NVP-BEZ235 and/or curcumin, 20 µl of XTT reaction solution (2,3-Bis(2-methoxy-4-nitro-5-sulfophenyl)-2H-tetrazolium-5-carboxanilide inner salt and phenazine methosulfate; mixed in proportion 50∶1) was added to the wells and measured by spectrophotometry at 450 nm with a microplate reader.

### Determination of Synergy

The possible synergistic effect of NVP-BEZ235 and curcumin was evaluated using the isobologram method. In brief, the cells were treated with different concentrations of NVP-BEZ235 and curcumin alone or in combination. After 48 h, relative survival was assessed and the concentration effect curves were used to determine the IC_50_ (the half-maximal inhibitory concentration) values for each drug alone and in combination with a fixed concentration of the second agent [48].

4′,6′-Diamidino-2-phenylindole staining for nuclear condensation and fragmentation.

To examine cellular nuclei, the cells were fixed with 1% paraformaldehyde on glass slides for 30 min at room temperature. After the fixation, the cells were washed with PBS and a 300 nM 4′,6′-diamidino-2-phenylindole solution (Roche, Mannheim, Germany) was added to the fixed cells for 5 min. After the nuclei were stained, the cells were examined by fluorescence microscopy.

### DNA Fragmentation Assay

The cell death detection ELISA plus kit (Boerhringer Mannheim; Indianapolis, IN) was used to determine the level of apoptosis by detecting fragmented DNA within the nuclei of NVP-BEZ235-treated cells, curcumin-treated cells, or cells that had been treated with a combination of NVP-BEZ235 and curcumin. Briefly, each culture plate was centrifuged for 10 min at 200 × *g*, the supernatant was removed, and the cell pellet was lysed for 30 min. Then, the plate was centrifuged again at 200 × *g* for 10 min, and the supernatant that contained the cytoplasmic histone-associated DNA fragments was collected and incubated with an immobilized anti-histone antibody. The reaction products were incubated with a peroxidase substrate for 5 min and measured by spectrophotometry at 405 and 490 nm (reference wavelength) with a microplate reader. The signals in the wells containing the substrate alone were considered as background and were subtracted.

### Asp-Glu-Val-Asp-ase (DEVDase) Activity Assay

To evaluate DEVDase activity, cell lysates of NVP-BEZ235-treated cells, curcumin-treated cells, or cells that were treated with a combination of NVP-BEZ235 and curcumin were prepared. Assays were performed in 96-well microtiter plates by incubating 20 µg cell lysate in 100 µl reaction buffer (1% NP-40, 20 mM Tris-HCl, pH 7.5, 137 mM NaCl, and 10% glycerol) containing a caspase substrate [Asp-Glu-Val-Asp-chromophore-p-nitroanilide (DVAD-pNA)] at 5 µM. Lysates were incubated at 37°C for 2 h, and the absorbance at 405 nm was measured with a spectrophotometer.

### Reverse Transcription Polymerase Chain Reaction (RT-PCR)

Total RNA was isolated using TRIzol reagent (Life Technologies; Gaithersburg, MD), and the cDNA was prepared using M-MLV reverse transcriptase (Gibco-BRL; Gaithersburg, MD) according to the manufacturer’s instructions. The following primers were used for the amplification of human Bcl-2, Mcl-1, and actin: Bcl-2 (sense) 5′- GGT GAA CTG GGG GAG GAT TGT-3′ and (antisense) 5′- CTT CAG AGA CAG CCA GGA GAA-3′; Mcl-1 (sense) 5′- GCG ACT GGC AAA GCT TGG CCT CAA-3′ and (antisense) 5′- GTT ACA GCT TGG ATC CCA ACT GCA-3′; and actin (sense) 5′- GGC ATC GTC ACC AAC TGG GAC -3′ and (anti-sense) 5′- CGA TTT CCC GCT CGG CCG TGG -3′. The PCR amplification was carried out using the following cycling conditions: 94°C for 3 min; followed by 17 (actin) or 23 cycles (Bcl-2 and Mcl-1) of 94°C for 45 s, 58°C for 45 s, and 72°C for 1 min; and a final extension at 72°C for 10 min. The amplified products were separated by electrophoresis on a 1.5% agarose gel and detected under UV light.

### Proteasome Activity Assay

Chymotryptic proteasome activities were measured with Suc-LLVY-AMC (chymotryptic substrate, Biomol International, Plymouth Meeting, PA). Lysate from NVP-BEZ235-treated cells was prepared. A mixture containing 1 µg cell lysate protein in 100 mM Tris-HCl (pH 8.0), 10 mM MgCl_2_, and 2 mM ATP was incubated at 37°C for 30 min with 50 µM Suc-LLVY-AMC. Enzyme activity was measured with a fluorometric plate reader at an excitation wavelength of 380 nm and an emission wavelength of 440 nm.

### Construction of Bcl-2 and Mcl-1 Stable Caki Cells

The Caki cells were stably transfected with pMAX-Bcl-2 (provided by Dr. Rakesh Srivastava, NIH/NIA), pcDNA3.1 Mcl-1 plasmid, or control plasmid pcDNA 3.1 vector using LipofectAMINE2000 as recommended by the manufacturer (Invitrogen). After 48 h of incubation, transfected cells were selected in cell culture medium containing 700 µg/ml G418 (Invitrogen). After 2 or 3 weeks, to eliminate the possibility of clonal differences between the generated stable cell lines, the pooled Caki/pcDNA 3.1 and Caki/Bcl-2, Caki/Mcl-1 clones were tested for Bcl-2 and Mcl-1 expression by immunoblotting, and the cells were used in this study.

### DNA Transfection and Luciferase Assay

Transient transfection was performed in 6-well plates. One day before the transfection, Caki cells were plated at approximately 60 to 80% confluence. The Bcl-2/−3254 promoter plasmid was transfected into the cells using Lipofectamine™ 2000 (Invitrogen; Carlsbad, CA). To assess the promoter-driven expression of the luciferase gene, the cells were collected and disrupted by sonication in lysis buffer (25 mM Tris-phosphate, pH 7.8, 2 mM EDTA, 1% Triton X-100, and 10% glycerol), and aliquots of the supernatant were used to analyze the luciferase activity according to the manufacturer's instructions (Promega; Madison, WI).

### Small Interfering RNA (siRNA)

The p53 siRNA duplexes used in this study were purchased from Santa Cruz Biotechnology (Santa Cruz, CA). Cells were transfected with siRNA oligonucleotides using Oligofectamine reagent (Invitrogen, Carlsbad, CA) according to the manufacturer's recommendations.

### Densitometry

The band intensities were scanned and quantified using the gel analysis plugin for the open source software ImageJ 1.46 (Imaging Processing and Analysis in Java; ttp://rsb.info.nih.gov/ij).

### Statistical Analysis

The data were analyzed using a one-way ANOVA and post-hoc comparisons (Student-Newman-Keuls) using the Statistical Package for Social Sciences 8.0 software (SPSS Inc.; Chicago, IL).

## Results

### NVP-BEZ235 has No Effect on Apoptosis in Human Renal Carcinoma Caki Cells

**Figure 1:**
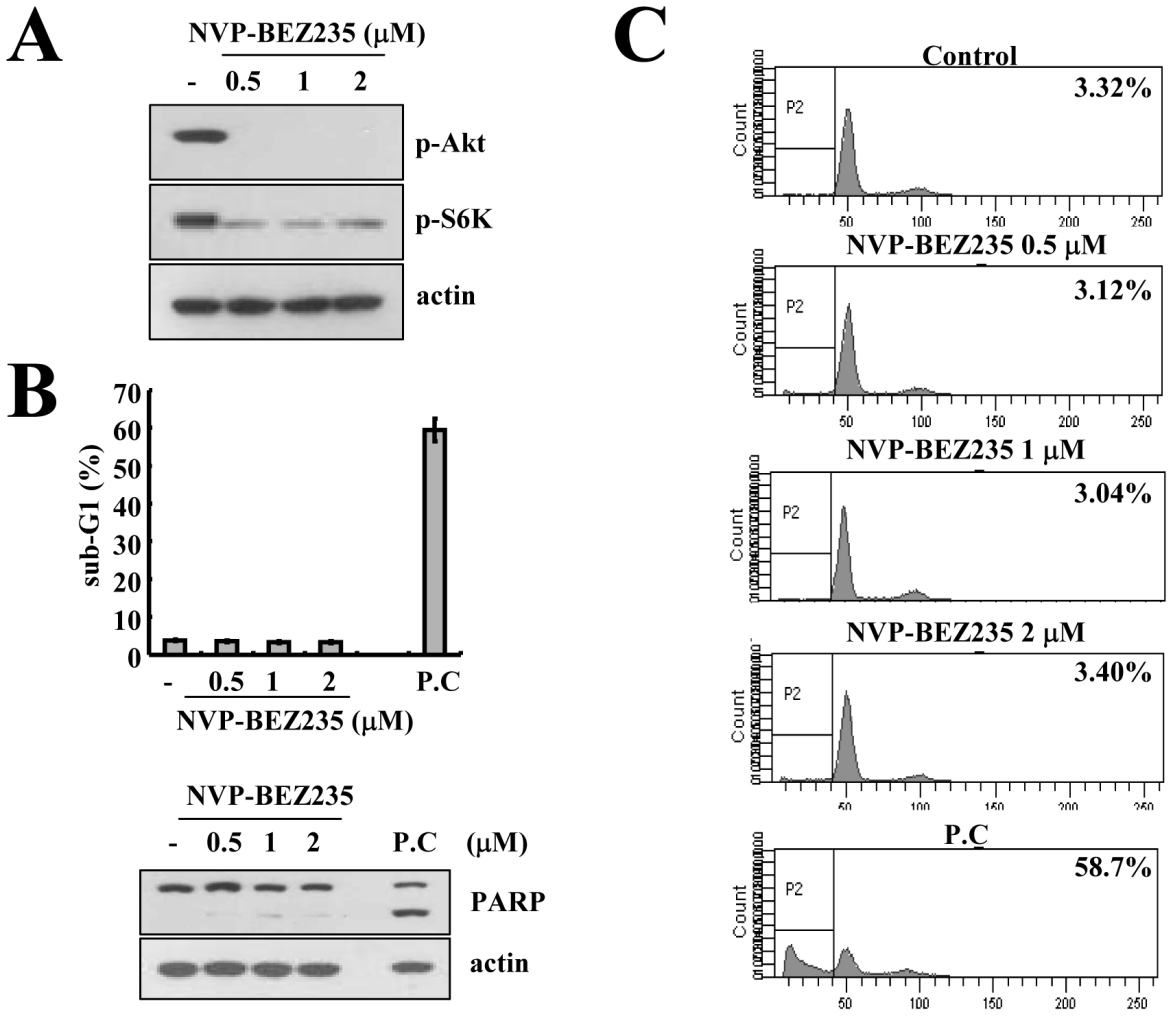
Effect of NVP-BEZ235 on apoptosis in human renal carcinoma Caki cells. Caki cells were treated with the indicated concentrations of NVP-BEZ235 for 48 h. (A) Equal amounts of cell lysate (40 µg) were subjected to electrophoresis and analyzed by western blotting for phospho (p)-Akt and p-S6K as well as actin as a control for protein loading. (B and C) Caki cells were treated with 100 nM TNF-α and 20 µg/ml cycloheximide for 48 h and used as a positive control (p.c.). The sub-G1 fraction was measured by flow cytometry (upper panel) as an indicator of the level of apoptosis. Equal amounts of cell lysate (40 µg) were subjected to electrophoresis and analyzed by western blotting for PARP and actin as a control for protein loading (lower panel). The values in (B) represent the mean ± SD from three independent samples. The data represent three independent experiments.

NVP-BEZ235 inhibits the activation of PI3K/Akt and mTOR signaling, which is important for cell survival, and consequently induces cell death in non-small lung cancer [49] and breast cancer cells [50]. Therefore, we tested whether NVP-BEZ235 induces cell death in renal carcinoma Caki cells. Although NVP-BEZ235 markedly inhibited Akt and S6K phosphorylation (Figure 1A), NVP-BEZ235 had no effect on the sub-G1 population and caspase-3-mediated PARP cleavage, which are markers of apoptosis, in Caki cells (Figure 1B and C).

### Synergistic Effect of Curcumin and NVP-BEZ235 on Apoptosis

**Figure 2:**
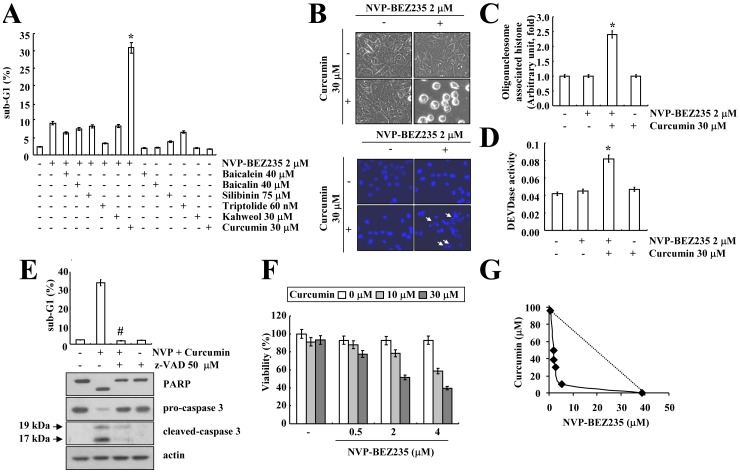
Synergistic effect of NVP-BEZ235 and curcumin on apoptosis in Caki cells. (A) Caki cells were treated with the indicated concentrations of baicalein, baicalin, silibinin, triptolide, kahweol, and curcumin in the absence or presence of 2 µM NVP-BEZ235 for 48 h. The sub-G1 fraction was measured by flow cytometry. (B–C) Caki cells were co-treated with 2 µM NVP-BEZ235 and 30 µM curcumin for 48 h. Cell morphology was detected by interference light microscopy (B, upper panel). The condensation and fragmentation of the nuclei were detected by 4′, 6′-diamidino-2-phenylindole staining (B, lower panel). The DNA fragmentation detection kit determined the fragmented DNA (C). Caspase activities were determined with colorimetric assays using caspase-3 DEVDase assay kits (D). (E) Caki cells were pretreated with 50 µM z-VAD-fmk (z-VAD) for 30 min, and then 2 µM NVP-BEZ235 plus 30 µM curcumin were added for 48 h. The sub-G1 fraction was measured by flow cytometry (upper panel) as an indicator of the level of apoptosis. Equal amounts of cell lysate (40 µg) were subjected to electrophoresis and analyzed by western blotting for PARP, pro-caspase 3, cleaved caspase-3 and actin as a control for protein loading (lower panel). (F) Caki cells were treated with the indicated concentrations of curcumin alone, NVP-BEZ235 alone or combined treatment with NVP-BEZ235 and curcumin for 48 h. The cell viability was assessed by XTT assay. (G) Isoboles were obtained by plotting the combined concentrations of each drug required to produce 50% cell death. The straight line connecting the IC_50_ values obtained for two agents when applied alone corresponds to an additivity of their independent effects. Values below this line indicate synergy, whereas values above this line indicate antagonism. The values in A, C, D, E, F, and G represent the mean ± SD from three independent samples. * *p*<0.001 compared to the NVP-BEZ235 alone and curcumin alone. # *p*<0.001 compared to the NVP-BEZ235 plus curcumin. The data represent three independent experiments.

Because natural compounds have anti-cancer potential, we investigated whether combined treatment with natural compounds and NVP-BEZ235 could modulate cell death in Caki cells. As shown in Figure 2A and Figure S3A, curcumin markedly increased the sub-G1 population in NVP-BEZ235-treated cells, but other compounds only had a minor effect on apoptosis. When we treated Caki cells with NVP-BEZ235 and/or curcumin, treatment with NVP-BEZ235 alone or curcumin alone did not cause morphological changes, but NVP-BEZ235 plus curcumin provoked cell shrinkage, membrane blebbing (Figure 2B, upper panel), and chromatin damage in the nuclei (Figure 2B, lower panel). DNA fragmentation was also increased by combined treatment with NVP-BEZ235 and curcumin (Figure 2C). Next, we determined whether apoptosis that is mediated by NVP-BEZ235 plus curcumin is dependent on caspase activation. Co-treatment with NVP-BEZ235 and curcumin markedly increased caspase activity and cleavage (Figure 2D–E). Moreover, z-VAD-fmk, a pan-caspase inhibitor, completely blocked the sub-G1 population, and PARP and caspase-3 cleavage in NVP-BEZ235 plus curcumin-treated cells (Figure 2E and Figure S3B). Next, we examined whether combined treatment with NVP-BEZ235 and curcumin have synergistic effects. Treatment with NVP-BEZ235 (0.5∼4 µM) alone had no effect on cell viability. However, combined treatment with a fixed concentrations of NVP-BEZ235 and varied concentrations of curcumin or with a fixed concentrations of curcumin and varied concentrations of NVP-BEZ235 markedly reduced cell viability (Figure 2F). The isobologram analysis suggested that combined treatment with NVP-BEZ235 and curcumin have synergistic effects (Figure 2G). Therefore, these data indicated that combined treatment with NVP-BEZ235 and curcumin induces apoptosis in a caspase-dependent manner, and have a synergistic effect in human renal Caki cells.

Combined treatment with NVP-BEZ235 and curcumin induces down-regulation of Mcl-1 and Bcl-2 expression.

**Figure 3:**
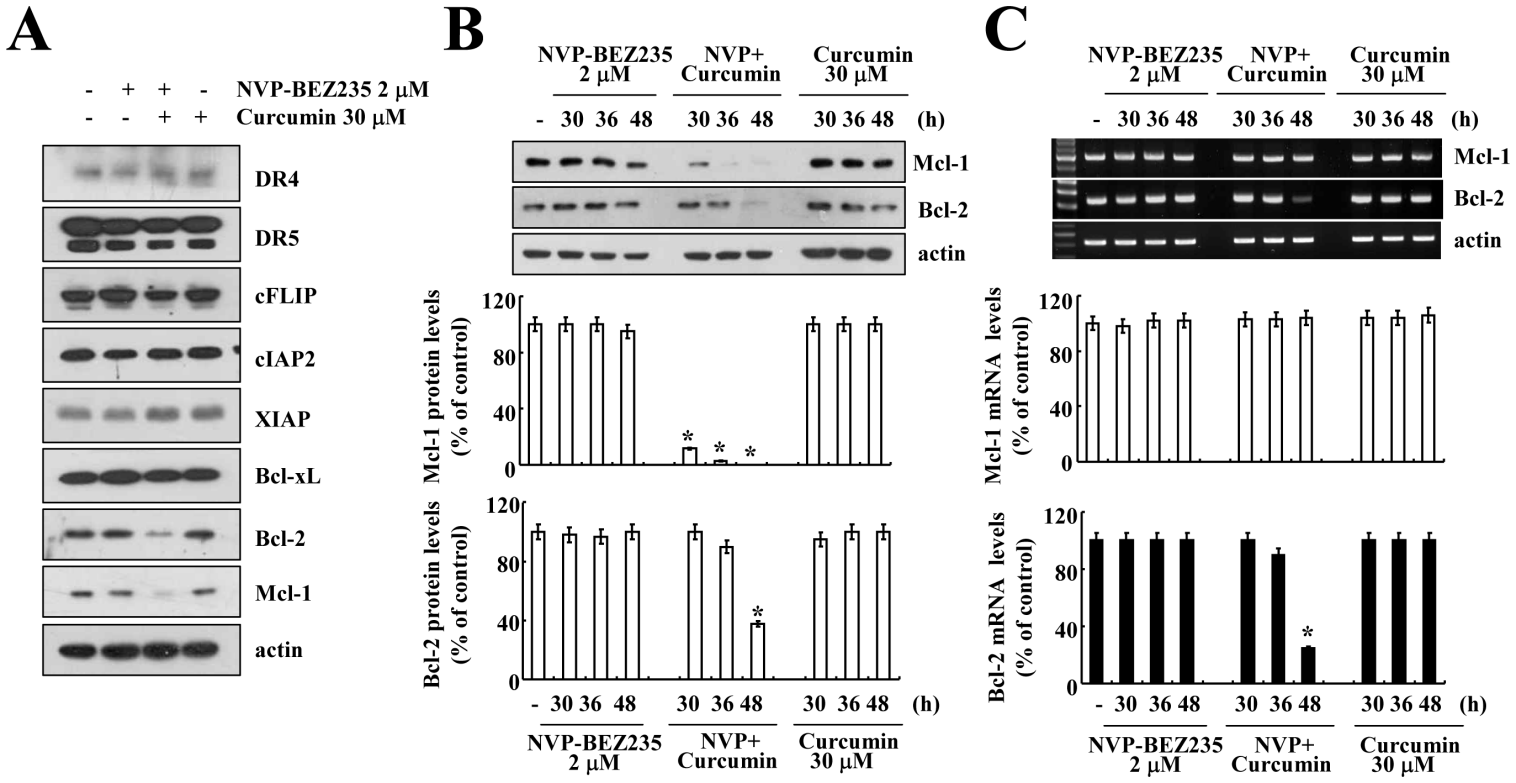
Effect of combined treatment with NVP-BEZ235 and curcumin on apoptosis-related proteins. (A) Caki cells were treated with 2 µM NVP-BEZ235 in the presence or absence of 30 µM curcumin for 48 h. Equal amounts of cell lysate (40 µg) were subjected to electrophoresis and analyzed by western blotting for DR4, DR5, cFLIP, cIAP2, XIAP, Bcl-xL, Bcl-2, and Mcl-1 and actin as a control for protein loading. (B and C) Caki cells were treated with 2 µM NVP-BEZ235 in the presence or absence of 30 µM curcumin for the indicated time periods. Equal amounts of cell lysate (40 µg) were subjected to electrophoresis and analyzed by western blotting for Mcl-1 and Bcl-2, as well as actin as a control for protein loading (B, upper panel). The Bcl-2 and Mcl-1 mRNA expression level was determined using RT-PCR (C, upper panel). The band intensities of Mcl-1 and Bcl-2 protein (B, upper panel) and mRNA (C, upper panel) were measured using the public domain JAVA image-processing program ImageJ (B and C, lower panel). The values in B and C represent the mean ± SD from three independent samples. * *p*<0.001 compared to NVP-BEZ235 alone and curcumin alone. The data represent three independent experiments.

Next, to identify the mechanism of NVP-BEZ235 plus curcumin-mediated apoptosis, we examined the expression of proteins that are associated with extrinsic (receptor-mediated) and intrinsic (mitochondria-mediated) apoptosis signaling. The expression levels of death receptors [(DR)4 and DR5], inhibitor of apoptosis protein (IAPs), and c-FLIP did not change in NVP-BEZ235 plus curcumin-treated cells. However, combined treatment with NVP-BEZ235 and curcumin, but not NVP-BEZ235 or curcumin alone, markedly reduced Mcl-1 and Bcl-2 expression (Figure 3A). The expression levels of Mcl-1 and Bcl-2 protein were down-regulated within 30 h and 48 h in NVP-BEZ235 plus curcumin-treated cells, respectively (Figure 3B). Furthermore, NVP-BEZ235 plus curcumin inhibited Bcl-2 mRNA expression, but Mcl-1 mRNA expression was unchanged (Figure 3B–C). Therefore, these data suggested that combined treatment with NVP-BEZ235 and curcumin modulates the expression of Mcl-1 at the post-translational level and Bcl-2 at the transcriptional level.

### Down-regulation of Mcl-1 is Involved in Combined Treatment with NVP-BEZ235 and Curcumin-induced Apoptosis

**Figure 4:**
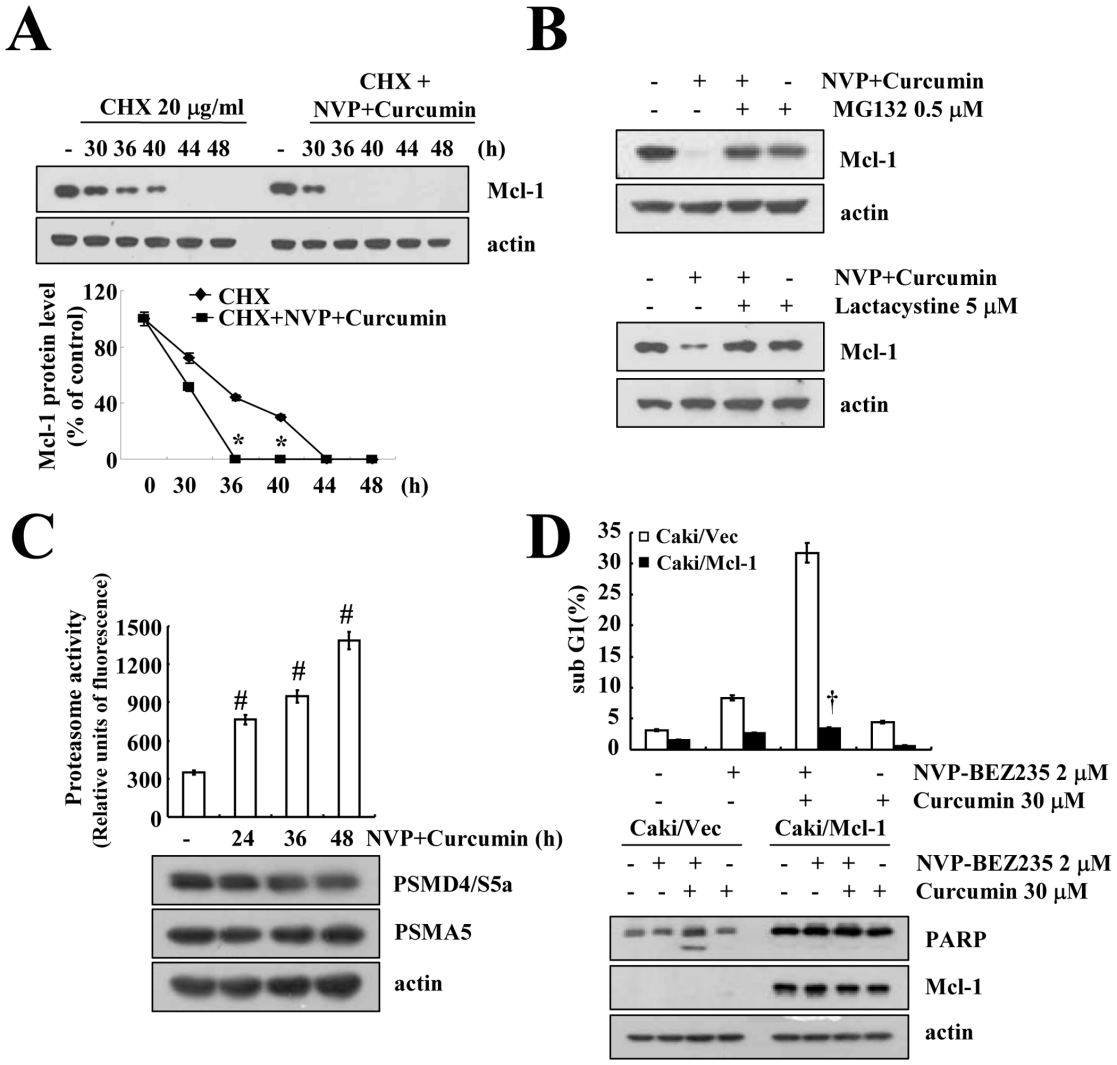
Combined treatment with NVP-BEZ235 and curcumin reduced Mcl-1 expression in a proteasome-dependent manner. (A) Caki cells were treated with or without 2 µM NVP-BEZ235 plus 30 µM curcumin in the presence of cycloheximide (CHX) (20 µg/ml) for the indicated time periods. The band intensities of Mcl-1 protein were measured using the public domain JAVA image-processing program ImageJ (lower panel). (B) Caki cells were pretreated with 0.5 µM MG132 and 5 µM lactacystin for 30 min, and then 2 µM NVP-BEZ235 plus 30 µM curcumin were added for 48 h. (C) Caki cells were treated with 2 µM NVP-BEZ235 plus 30 µM curcumin for the indicated time periods. The cells were lysed, and proteasome activity was measured as described in the Materials and Methods section. (D) Cells that were transfected with the empty vector (Caki/Vec) and cells that over-expressed Mcl-1 (Caki/Mcl-1) were treated with 2 µM NVP-BEZ235 in the presence or absence of 30 µM curcumin for 48 h. Equal amounts of cell lysate (40 µg) were subjected to electrophoresis and analyzed by western blotting for Mcl-1 (A, B, and D, lower panel), PSMD4/S5a (C), PSMA5 (C), and PARP (D), as well as actin as a control for protein loading. The sub-G1 fraction was measured by flow cytometry (D, upper panel) as an indicator of the level of apoptosis. The values in A, C, and D represent the mean ± SD from three independent samples. * *p*<0.001 compared to CHX alone. # *p*<0.001 compared to control. † *p*<0.001 compared to Caki/Vec, which combined treatment with 2 µM NVP-BEZ235 plus 30 µM curcumin. The data represent three independent experiments.

Because combined treatment with NVP-BEZ235 and curcumin had no effect on Mcl-1 mRNA expression, we subsequently examined the protein stability of Mcl-1. Caki cells were treated with or without NVP-BEZ235 plus curcumin in the presence of cycloheximide (CHX) (20 µg/ml) for various time points. As shown in Figure 4A, combined treatment with NVP-BEZ235 and curcumin reduced the stability of the Mcl-1 protein. Because Mcl-1 is mainly degraded by the proteasome [51], we tested the effect of proteasome inhibitors (MG132 and lactacystin) on Mcl-1 degradation. Proteasome inhibitors completely reversed the NVP-BEZ235 plus curcumin-mediated down-regulation of Mcl-1 (Figure 4B). Furthermore, NVP-BEZ235 plus curcumin markedly increased proteasome activity (Figure 4C). We further examined whether NVP-BEZ235 plus curcumin induces the protein expression of two critical proteasome subunits [20S proteasome subunit alpha type 5 (PSMA5) and 19S proteasome non-ATPase regulatory subunit 4 (PSMD4/S5a)] [52], but the up-regulation of proteasome activity was not associated with PSMA5 and PSMD4/S5a expression in the NVP-BEZ235 plus curcumin-treated Caki cells (Figure 4C). To confirm the functional significance of Mcl-1 down-regulation, Mcl-1 protein was over-expressed in Caki cells. As expected, when Mcl-1 was over-expressed, we observed a significant decrease in apoptosis and PARP cleavage in NVP-BEZ235 plus curcumin-treated cells (Figure 4D and Figure S4A).

### Down-regulation of Bcl-2 is Involved in Combined Treatment with NVP-BEZ235 and Curcumin-induced Apoptosis

**Figure 5:**
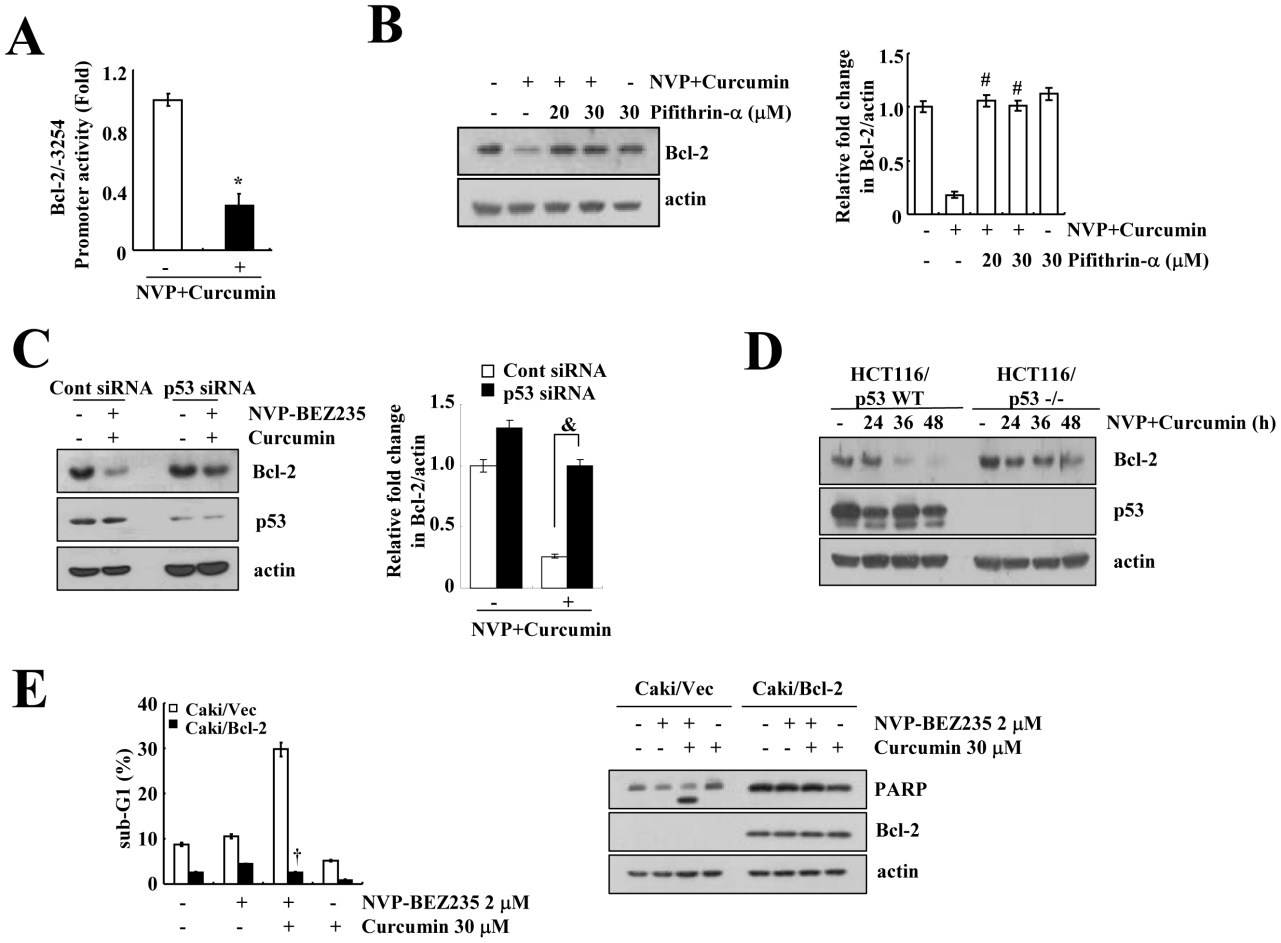
Combined treatment with NVP-BEZ235 (NVP) and curcumin decreased Bcl-2 expression in a p53-dependent manner. (A) Caki cells were transiently transfected with a plasmid harboring the luciferase gene under the control of the Bcl-2/−3254 promoter. After transfection, the Caki cells were treated with 2 µM NVP-BEZ235 plus 30 µM curcumin for 48 h. After treatment, the cells were lysed, and the luciferase activity was analyzed. (B) Caki cells were pretreated with pifithrin-α for 30 min and were then treated with 2 µM NVP-BEZ235 plus 30 µM curcumin for 48 h. (C) Caki cells were transiently transfected with a control (Cont. siRNA) or p53 siRNA. Twenty-four hours after transfection, cells were treated with 2 µM NVP-BEZ235 plus 30 µM curcumin for 48 h. (D) p53 wild-type (HCT116/p53+/+) and p53-null HCT116 cells (HCT116/p53−/−) were treated with 2 µM NVP-BEZ235 and 30 µM curcumin for the indicated time periods. (E) Cells that were transfected with the empty vector (Caki/Vec) and cells that over-expressed Bcl-2 (Caki/Bcl-2) were treated with 2 µM NVP-BEZ235 plus 30 µM curcumin for 48 h. The sub-G1 fraction was measured by flow cytometry. Equal amounts of cell lysate (40 µg) were subjected to electrophoresis and analyzed by western blotting for Bcl-2 (B, C, D, and E), p53 (C and D), PARP (E), and actin as a control for protein loading. The band intensities of Bcl-2 protein were measured using the public domain JAVA image-processing program ImageJ (B and C). The values in A, B, C, and E represent the mean ± SD from three independent samples. * *p*<0.001 compared to control. # *p*<0.001 compared to 2 µM NVP-BEZ235 and 30 µM curcumin. & *p*<0.001 compared to cont siRNA, which combined treated with 2 µM NVP-BEZ235 and 30 µM curcumin. † *p*<0.001 compared to Caki/Vec, which combined treated with 2 µM NVP-BEZ235 plus 30 µM curcumin. The data represent three independent experiments.

Next, we examined the mechanism of Bcl-2 down-regulation in NVP-BEZ235 plus curcumin-treated cells. Combined treatment with NVP-BEZ235 and curcumin led to a reduction in Bcl-2 protein expression at the transcriptional level (Figure 3B–C). To confirm these data, Caki cells were transfected with a Bcl-2 promoter (Bcl-2/−3254) construct. The promoter activity of Bcl-2 was markedly reduced in NVP-BEZ235- and curcumin-treated cells (Figure 5A). Among the regulatory mechanisms of Bcl-2 expression at the transcriptional level, we found that pifithrin-α, a p53 inhibitor, reversed the NVP-BEZ235 plus curcumin-mediated down-regulation of Bcl-2 expression (Figure 5B). Furthermore, the down-regulation of p53 expression by p53 siRNA also overcame the down-regulation of Bcl-2 expression (Figure 5C). To further determine the role of p53 on the down-regulation of Bcl-2 expression, we compared the response to NVP-BEZ235 plus curcumin in isogenic human colon carcinoma cell lines differing only in the presence or absence of p53 [HCT116/p53(+/+) and HCT116/p53(−/−)]. Combined treatment with NVP-BEZ235 and curcumin had no effect on Bcl-2 expression in HCT116/p53 (−/−) cells, while Bcl-2 expression was dramatically reduced in HCT116/p53 (+/+) cells (Figure 5D). Therefore, these results indicated that the combination of NVP-BEZ235 and curcumin down-regulates Bcl-2 expression through the activation of p53. Next, to verify the functional importance of reduced Bcl-2 expression, we determined the effect of combined treatment with NVP-BEZ235 and curcumin on apoptosis in Bcl-2-over-expressing cells (Caki/Bcl-2). As shown in Figure 5E and Figure S4B, ectopic expression of Bcl-2 blocked the induction of the sub-G1 population and PARP cleavage in NVP-BEZ235 plus curcumin-treated cells.

Effects of combined treatment with NVP-BEZ235 and curcumin on the other renal carcinoma cells and normal cells.

**Figure 6:**
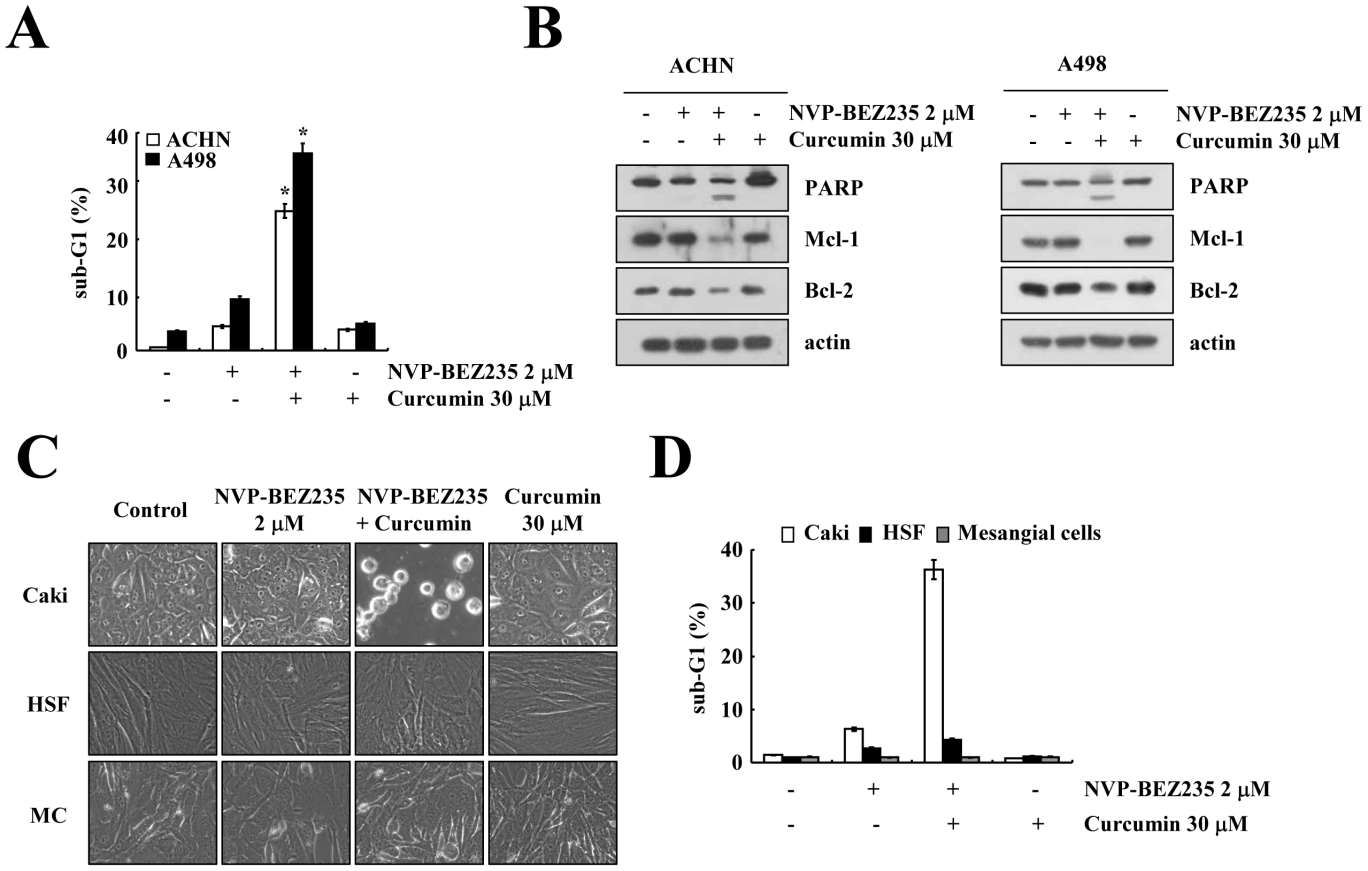
Effect of combined treatment with NVP-BEZ235 (NVP) and curcumin on apoptosis in other renal cancer cells and normal cells. Other renal cancer cells (ACHN and A498) and normal cells (HSFs and MCs) were treated with 2 µM NVP-BEZ235 plus 30 µM curcumin for 48 h. The sub-G1 fraction was measured by flow cytometry (A and D). Equal amounts of cell lysate (40 µg) were subjected to electrophoresis and analyzed by western blotting for PARP, Mcl-1, and Bcl-2 and actin as a control for protein loading (B). Cell morphology was detected by interference light microscopy (C). * *p*<0.001 compared to NVP-BEZ235 alone and curcumin alone. The values in A and D represent the mean ± SD from three independent samples. The data represent three independent experiments.

We investigated whether NVP-BEZ235 and curcumin could enhance apoptosis in other renal carcinoma cell lines (ACHN and A498) and normal cells [human skin fibroblasts (HSFs) and mouse mesangial cells (MCs)]. We found that combined treatment with NVP-BEZ235 and curcumin enhanced the sub-G1 population and PARP cleavage in ACHN and A498 cells (Figure 6A–B, and Figure S5A). Furthermore, the expression of Mcl-1 and Bcl-2 was reduced in NVP-BEZ235 plus curcumin-treated cells (Figure 6B). Combined treatment with NVP-BEZ235 and curcumin also induced the sub-G1 population and PARP cleavage in other cells [breast carcinoma (MDA-MB231) and glioma (U87MG) cells] (Figure S1). In contrast, combined treatment with NVP-BEZ235 and curcumin had no effect on apoptosis in HSFs and MCs (Figure 6C–D, Figure S2, and Figure S5B).

**Figure 7:**
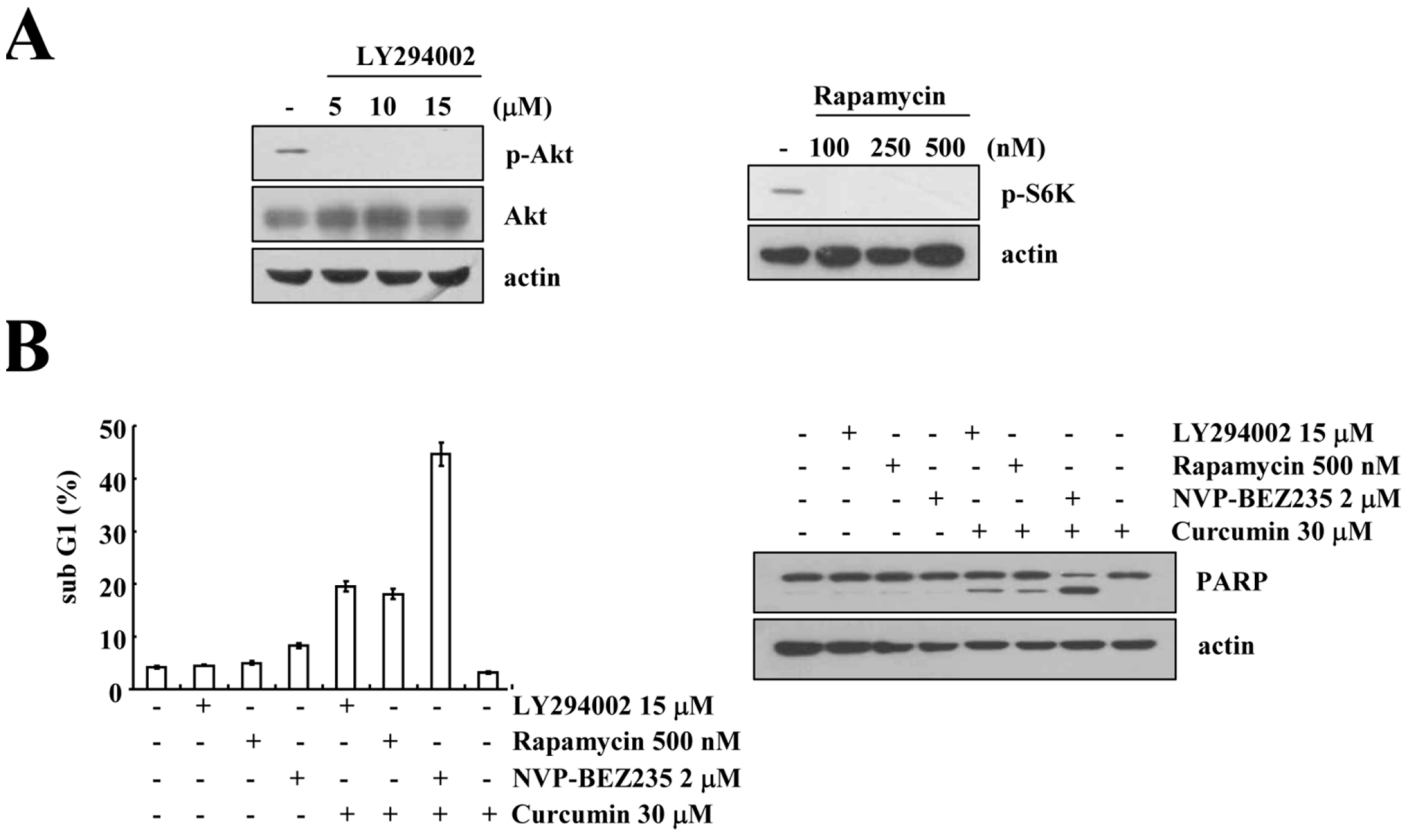
Effect of rapamycin, LY294002, and NVP-BEZ235 on apoptosis in curcumin-treated cells. (A) Caki cells were treated with the indicated concentrations of LY294002 (left panel) and rapamycin (right panel) for 6 h. Equal amounts of cell lysate (40 µg) were subjected to electrophoresis and analyzed by western blotting for phospho (p)-Akt, Akt, and p-S6K, as well as actin as a control for protein loading. (B) Caki cells were treated with the indicated concentrations of LY294002, rapamycin, and NVP-BEZ235 in the absence or presence of 30 µM curcumin for 48 h. The sub-G1 fraction was measured by flow cytometry. Equal amounts of cell lysate (40 µg) were subjected to electrophoresis and analyzed by western blotting for PARP and actin as a control for protein loading. The values in (B) represent the mean ± SD from three independent samples. The data represent three independent experiments.

Finally, we examined whether the effect of NVP-BEZ235 on apoptosis is comparable to that of PI3K/Akt inhibitor (LY294002) and mTORC1 inhibitor (rapamycin) treatment in curcumin-treated cells. LY294002 and rapamycin markedly inhibited the phosphorylation of Akt and S6K, respectively (Figure 7A). Combined treatment with NVP-BEZ235 and curcumin had the strongest effect on the induction of apoptosis (Figure 7B and Figure S6). These data indicated that dual PI3K/Akt and mTOR inhibition is more effective for improving the anti-cancer effect.

Taken together, our results demonstrate that combined treatment with NVP-BEZ235 and curcumin has a synergistic effect on apoptosis in human renal carcinoma cells via proteasome-dependent Mcl-1 protein degradation and p53-dependent inhibition of Bcl-2 expression.

## Discussion

In this study, we demonstrated that NVP-BEZ235 plus curcumin have a synergistic effect on apoptosis in human carcinoma Caki cells through the modulation of Mcl-1 protein stability and the inhibition of Bcl-2 expression in a p53-dependent manner. Furthermore, combined treatment with NVP-BEZ235 and curcumin also induced apoptosis in other renal carcinoma cells (ACHN and A498), but not normal cells (HSFs and MCs). Therefore, our results suggest that combined treatment with NVP-BEZ235 and curcumin is a more efficient and safe strategy to induce cancer cell death.

Bcl-2 is an anti-apoptotic protein, and the down-regulation of Bcl-2 is important for the induction of apoptosis by anti-cancer drugs. The expression of Bcl-2 is modulated at the transcriptional and/or post-transcriptional levels. Two promoters, including P1 and P2, are key control sites that modulate Bcl-2 expression [53]. P1 promoter is major promoter, and it is located 715 bp upstream of the Bcl-2 transcriptional start site. The P1 promoter is a GC-rich and TATA-less promoter. It contains multiple transcription initiation sites and includes seven Sp1 binding sites [53]. The P2 promoter is located 1.3 kb downstream of the P1 promoter, and it contains TATA and CAAT boxes. The positive regulation of Bcl-2 transcription is modulated by cAMP responsive element binding protein [54], C/EBP [55], and NF-κB [56]. In addition, Bcl-2 is also negatively regulated by π1 [57], WT1 [58], and p53 [59]. In our study, p53 was involved in the down-regulation of Bcl-2 expression in NVP-BEZ235 plus curcumin-treated cells (Figure 5B–D). Although combined treatment with NVP-BEZ235 and curcumin did not increase p53 expression (Figure 5C), pifithrin-α (p53 inhibitor) and the down-regulation of p53 by p53 siRNA markedly reversed the effect of the combined treatment with NVP-BEZ235 and curcumin-mediated down-regulation of Bcl-2 expression (Figure 5B–C). Furthermore, Bcl-2 expression did not change due to combined treatment with NVP-BEZ235 and curcumin in p53 null HCT116 cells (HCT116/p53−/−). Therefore, p53 is a key regulator to reduce Bcl-2 expression in NVP-BEZ235 plus curcumin-treated cells.

PI3K/Akt and mTOR are well-known major regulatory signaling pathways that modulate cell survival in cancer cells. Therefore, several inhibitors, that block these signaling pathways have been developed and used for treatment to increase apoptosis in cancer cells. It has been previously demonstrated that LY294002 and wortmannin, which inhibit the activation of PI3K/Akt signaling, are effective compounds against tumors in vivo [22], [60]. However, the PI3K/Akt signaling pathway also regulates physiological response, such as insulin action. Thus, it is risky to use a broad spectrum inhibitor of PI3K/Akt signaling, and its chronic administration is very dangerous [61]. To inhibit the mTOR signaling pathway, many researchers have used rapamycin. However, rapamycin only inhibits mTOR complex (TORC) 1, and it consequently induces Akt phosphorylation via feedback activation [18]. These actions reduce the anti-cancer effect of rapamycin. NVP-BEZ235 inhibits PI3K/Akt and mTOR (TORC1 and TORC2); thus, it efficiently reduces tumor growth in in vivo models [62], [63]. However, NVP-BEZ235 is a reversible inhibitor; thus, in vivo Akt phosphorylation is reversed at 24 h [25]. Therefore, tumor cells can be treated repeatedly with NVP-BEZ235. However, when cells were co-treated with NVP-BEZ235 and curcumin, inhibition of Akt and S6K phosphorylation was maintained at 48 h (data not shown). Although curcumin alone did not inhibit PI3K/Akt and mTOR signaling under the conditions that we used, curcumin could help NVP-BEZ235 to act as an inhibitor of PI3K/Akt and mTOR. Further experiments are required to verify curcumin’s mechanism of action. In our study, although NVP-BEZ235 markedly inhibited Akt and S6K phosphorylation, NVP-BEZ235 was not sufficient to induce apoptosis in human renal Caki cells (Figure 1). Furthermore, LY294002 and rapamycin inhibited the phosphorylation of Akt and S6K, respectively, but they did not induce apoptosis (Figure 7). Thus, we developed a combined treatment strategy using a natural compound, curcumin. Combined treatment with NVP-BEZ235 and curcumin has a synergistic effect on the induction of apoptosis. NVP-BEZ235 induced apoptosis more effectively in curcumin-treated cells, compared with LY294002 or rapamycin (Figure 7). These findings probably result from the dual PI3K/Akt and mTOR inhibition of NVP-BEZ235.

Curcumin has been known as an extremely safe compound at high dose. Lao et al., reported although healthy volunteers were administered doses from 500 to 12,000 mg, only 30% of volunteers had minimal toxicity that was not related with dose of curcumin [64]. For chemoprevention in human, curcumin is required daily 1.6 g/person [65]. However, it is not pharmacokinetically possible to take the human blood volume and calculate concentration of drug on the basis of the dose ingested. Clinical trials of oral curcumin cannot be utilized because of low bioavailability, poor absorption, rapid elimination and/or low target organ concentration. However, adjuvant could increase curcumin bioavailability through inhibition of metabolism. Co-ingestion of curcumin with piperine, which is a inhibitor of hepatic and intestinal glucuronidation, appeared 2000% increase bioavailability of curcumin [66]. In addition, micronized powder and liquid micelles markedly induced 9-folds and 185-folds better bioavailability than native curcumin, respectively [67].

Taken together, our results suggest that combined treatment with NVP-BEZ235 and curcumin could be an effective anti-cancer therapy.

## Supporting Information

Figure S1 **Synergistic effect of NVP-BEZ235 and curcumin on apoptosis in MDA-MB231 and U87MG cells.** MDA-MB231 (A) and U87MG cells (B) were treated with 2 µM NVP-BEZ235 plus 30 µM curcumin for 48 h. The sub-G1 fraction was measured by flow cytometry (upper panel). Equal amounts of cell lysate (40 µg) were subjected to electrophoresis and analyzed by western blotting for PARP and actin as a control for protein loading (lower panel). The values represent the mean ± SD from three independent samples. * p<0.001 compared to the NVP-BEZ235 alone and curcumin alone. The data represent three independent experiments.
(TIF)

Figure S2 **Effect of NVP-BEZ235 on apoptosis in normal cells [human skin fibroblasts (HSF) and mouse mesangial cells (MC)].** HSF, MC and Caki cells were co-treated with 2 µM NVP-BEZ235 plus 30 µM curcumin for 48 h. (A) The condensation and fragmentation of the nuclei were detected by 4′,6′-diamidino-2-phenylindole staining (B) The DNA fragmentation detection kit determined the fragmented DNA. (C) Caspase activities were determined with colorimetric assays using caspase-3 DEVDase assay kits. The values in B and C represent the mean ± SD from three independent samples. The data represent three independent experiments.
(TIF)

Figure S3 **Histograms of** **Fig. 2A** **(A) and** **Fig. 2E** **(B).** The sub-G1 fraction was measured by flow cytometry. Histograms of Fig. 2A (A) and Fig. 2E (B).
(TIF)

Figure S4 **Histograms of** **Fig. 4D** **(A) and** **Fig. 5E** **(B).** The sub-G1 fraction was measured by flow cytometry. Histograms of Fig. 4D (A) and Fig. 5E (B).
(TIF)

Figure S5 **Histograms of** **Fig. 6A** **(A) and** **Fig. 6D** **(B).** The sub-G1 fraction was measured by flow cytometry. Histograms of Fig. 6A (A) and Fig. 6D (B).
(TIF)

Figure S6 **Histograms of** **Fig. 7B** **.** The sub-G1 fraction was measured by flow cytometry. Histograms of Fig. 7B.
(TIF)
