# Correction: Curcumin Significantly Enhances Dual PI3K/Akt and mTOR Inhibitor NVP-BEZ235-Induced Apoptosis in Human Renal Carcinoma Caki Cells through Down-Regulation of p53-Dependent Bcl-2 Expression and Inhibition of Mcl-1 Protein Stability

**DOI:** 10.1371/journal.pone.0151886

**Published:** 2016-03-14

**Authors:** Bo Ram Seo, Kyoung-jin Min, Il Je Cho, Sang Chan Kim, Taeg Kyu Kwon

The authors would like to correct Fig 2, as errors were introduced in the preparation of the figure for publication. In the lower panel of Fig 2B, the Curcumin 30 μM + panel and the NVP-BEZ235 2μM + panel are erroneously derived from the same image. A corrected version of Fig 2 is available here. The authors confirm that these changes to not alter their findings and have provided the underlying images as Supporting Information.

**Fig 2:**
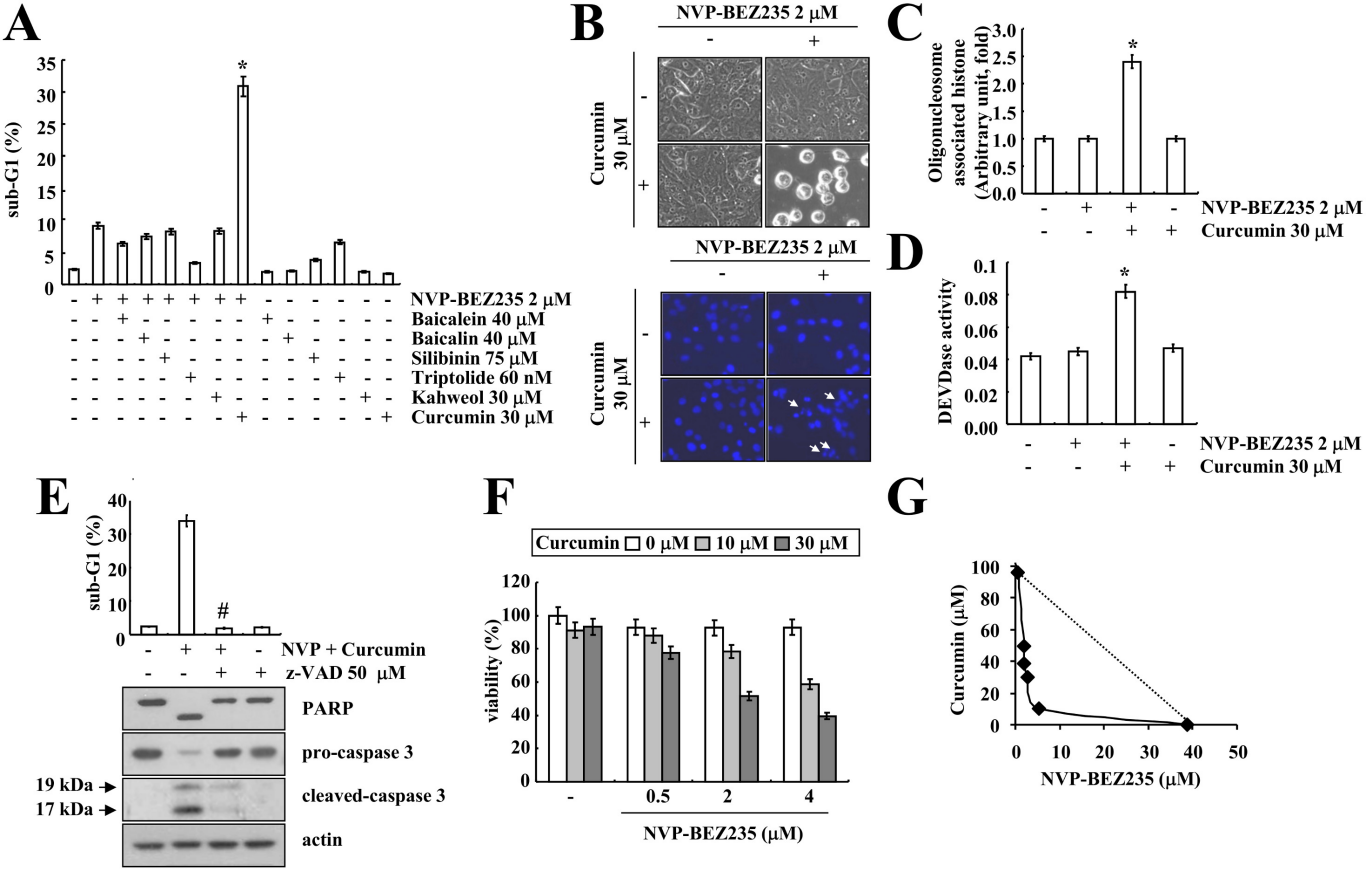
Synergistic effect of NVP-BEZ235 and curcumin on apoptosis in Caki cells. (A) Caki cells were treated with the indicated concentrations of baicalein, baicalin, silibinin, triptolide, kahweol, and curcumin in the absence or presence of 2 μM NVP-BEZ235 for 48 h. The sub-G1 fraction was measured by flow cytometry. (B–C) Caki cells were co-treated with 2 μM NVP-BEZ235 and 30 μM curcumin for 48 h. Cell morphology was detected by interference light microscopy (B, upper panel). The condensation and fragmentation of the nuclei were detected by 4′, 6′-diamidino-2-phenylindole staining (B, lower panel). The DNA fragmentation detection kit determined the fragmented DNA (C). Caspase activities were determined with colorimetric assays using caspase-3 DEVDase assay kits (D). (E) Caki cells were pretreated with 50 μM z-VAD-fmk (z-VAD) for 30 min, and then 2 μM NVP-BEZ235 plus 30 μM curcumin were added for 48 h. The sub-G1 fraction was measured by flow cytometry (upper panel) as an indicator of the level of apoptosis. Equal amounts of cell lysate (40 μg) were subjected to electrophoresis and analyzed by western blotting for PARP, pro-caspase 3, cleaved caspase-3 and actin as a control for protein loading (lower panel). (F) Caki cells were treated with the indicated concentrations of curcumin alone, NVP-BEZ235 alone or combined treatment with NVP-BEZ235 and curcumin for 48 h. The cell viability was assessed by XTT assay. (G) Isoboles were obtained by plotting the combined concentrations of each drug required to produce 50% cell death. The straight line connecting the IC_50_ values obtained for two agents when applied alone corresponds to an additivity of their independent effects. Values below this line indicate synergy, whereas values above this line indicate antagonism. The values in A, C, D, E, F, and G represent the mean ± SD from three independent samples. * *p*<0.001 compared to the NVP-BEZ235 alone and curcumin alone. # *p*<0.001 compared to the NVP-BEZ235 plus curcumin. The data represent three independent experiments.

## Supporting Information

S1 File Raw images used to create Fig 2. (PPT)
