# Supplementary figures and images for: Curcumin Significantly Enhances Dual PI3K/Akt and mTOR Inhibitor NVP-BEZ235-Induced Apoptosis in Human Renal Carcinoma Caki Cells through Down-Regulation of p53-Dependent Bcl-2 Expression and Inhibition of Mcl-1 Protein Stability
Source: PLoS One. 2014 Apr 17;9(4):e95588. doi: 10.1371/journal.pone.0095588 (PMC3990719; doi:10.1371/journal.pone.0095588)

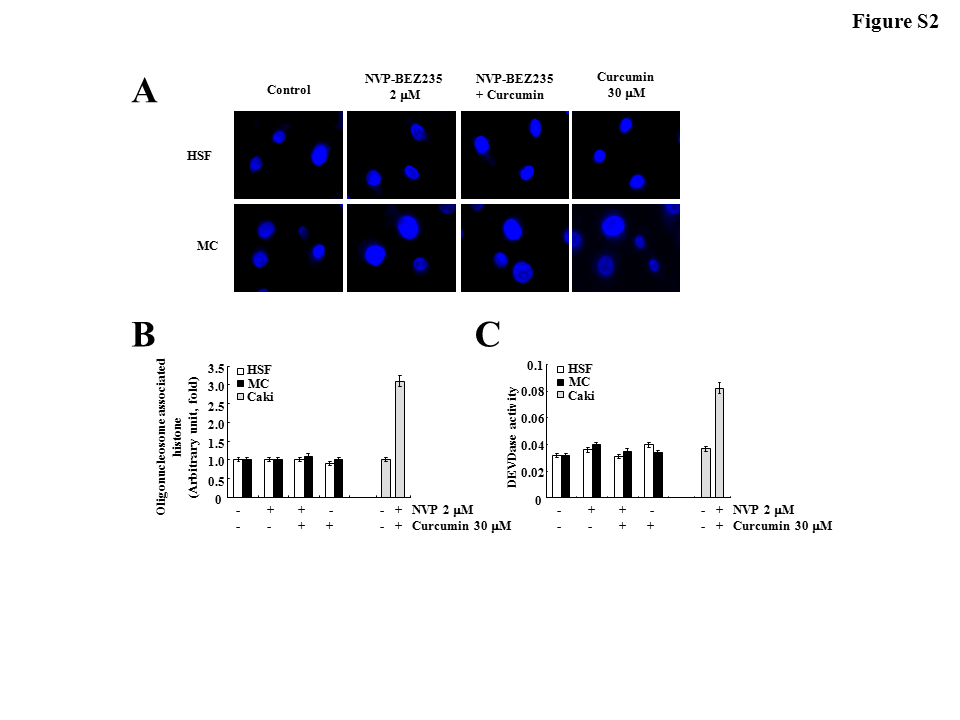

Supplement: Figure S2 — Effect of NVP-BEZ235 on apoptosis in normal cells [human skin fibroblasts (HSF) and mouse mesangial cells (MC)]. HSF, MC and Caki cells were co-treated with 2 µM NVP-BEZ235 plus 30 µM curcumin for 48 h. (A) The condensation and fragmentation of the nuclei were detected by 4′,6′-diamidino-2-phenylindole staining (B) The DNA fragmentation detection kit determined the fragmented DNA. (C) Caspase activities were determined with colorimetric assays using caspase-3 DEVDase assay kits. The values in B and C represent the mean ± SD from three independent samples. The data represent three independent experiments. (TIF) [file pone.0095588.s002.tif]
