# Supplementary material for: Correction: Curcumin Significantly Enhances Dual PI3K/Akt and mTOR Inhibitor NVP-BEZ235-Induced Apoptosis in Human Renal Carcinoma Caki Cells through Down-Regulation of p53-Dependent Bcl-2 Expression and Inhibition of Mcl-1 Protein Stability
Source: PLoS One. 2016 Mar 14;11(3):e0151886. doi: 10.1371/journal.pone.0151886 (PMC4790885; doi:10.1371/journal.pone.0151886)
Supplement: S1 File — (PPT) [file pone.0151886.s001.ppt]

## Slide 1
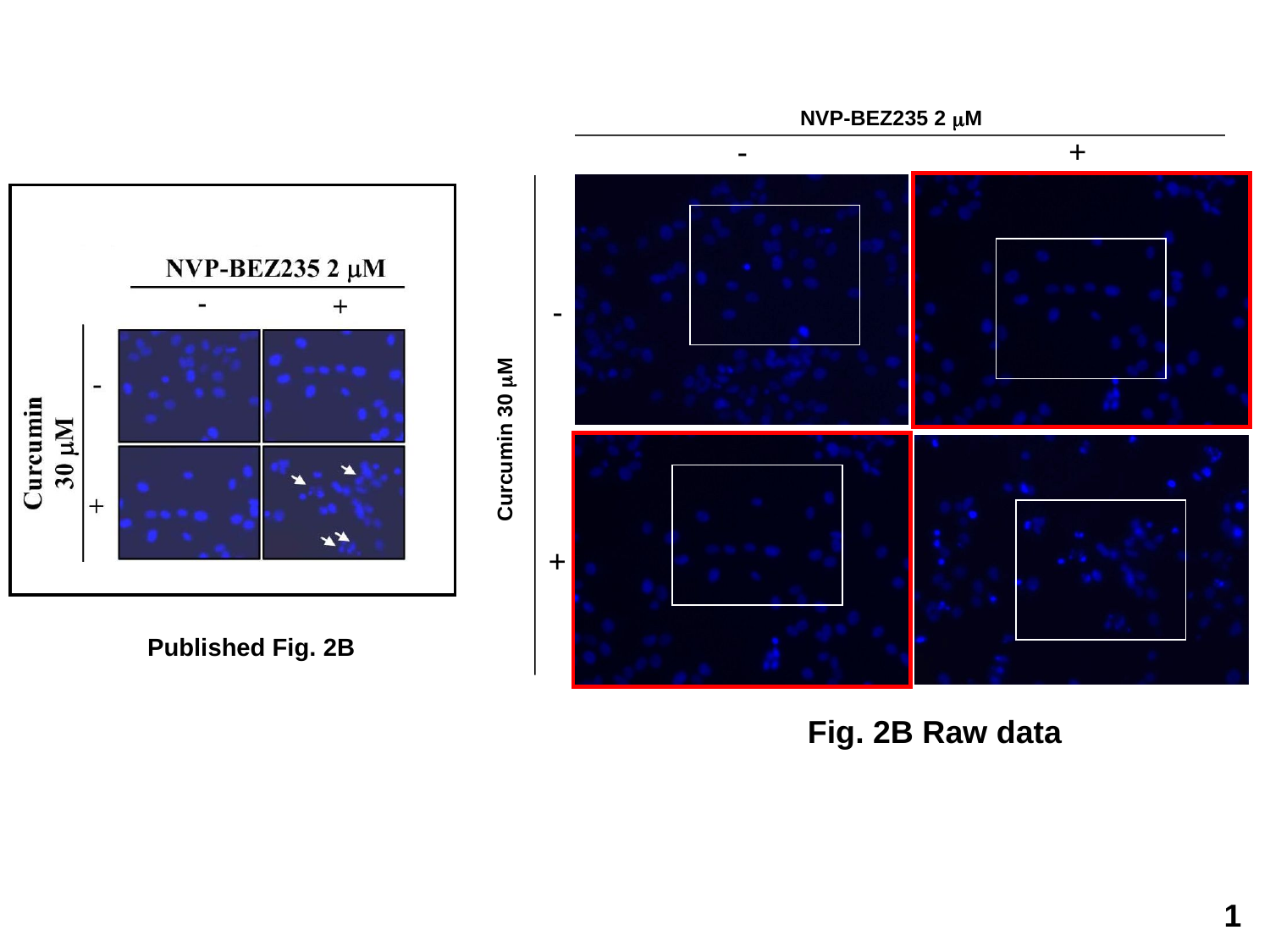

NVP-BEZ235 2 μM
-
+
-
Curcumin 30 μM
+
Published Fig. 2B
Fig. 2B Raw data
1

## Slide 2
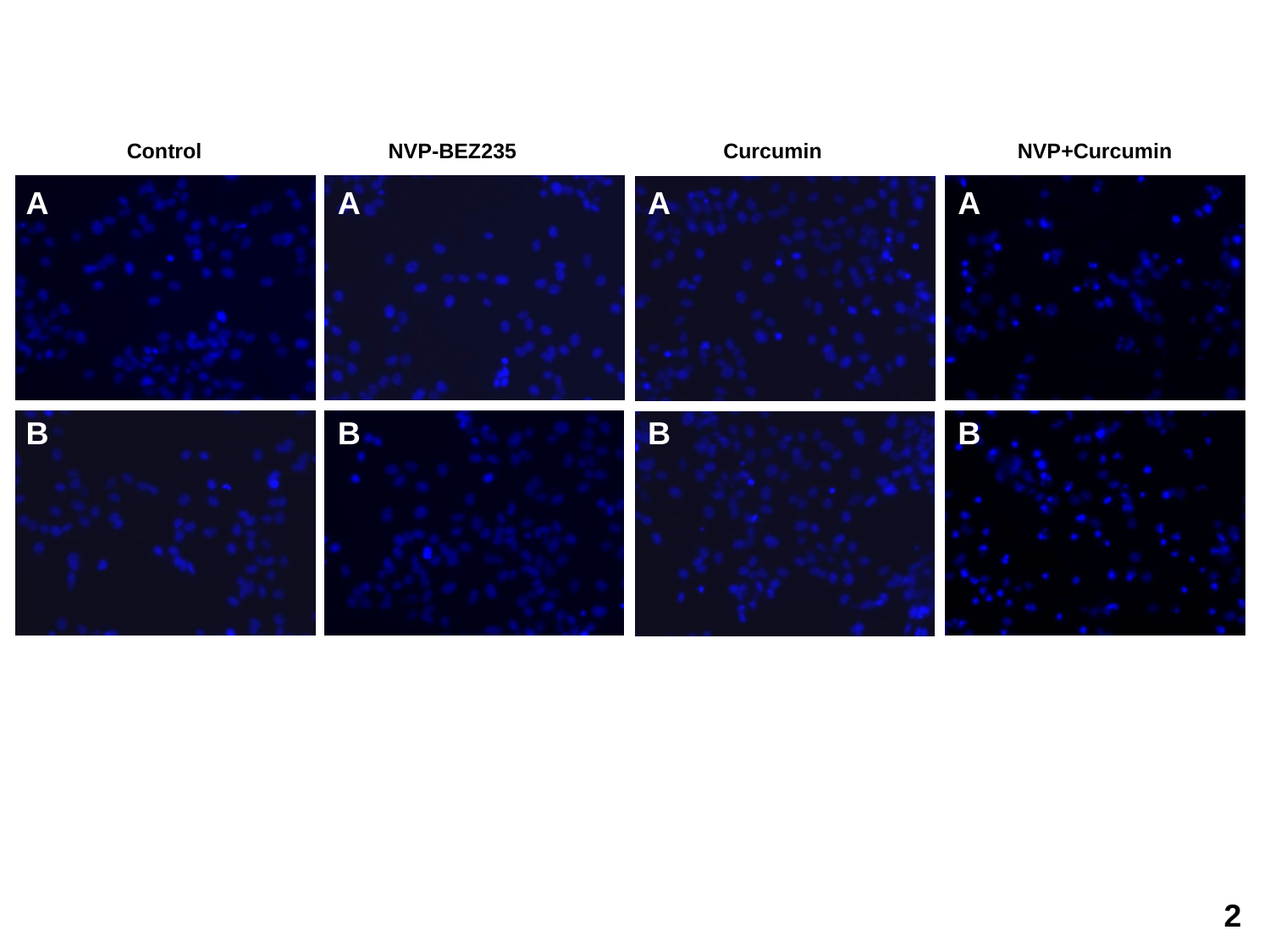

Control
NVP-BEZ235
Curcumin
NVP+Curcumin
A
A
A
A
B
B
B
B
2

## Slide 3
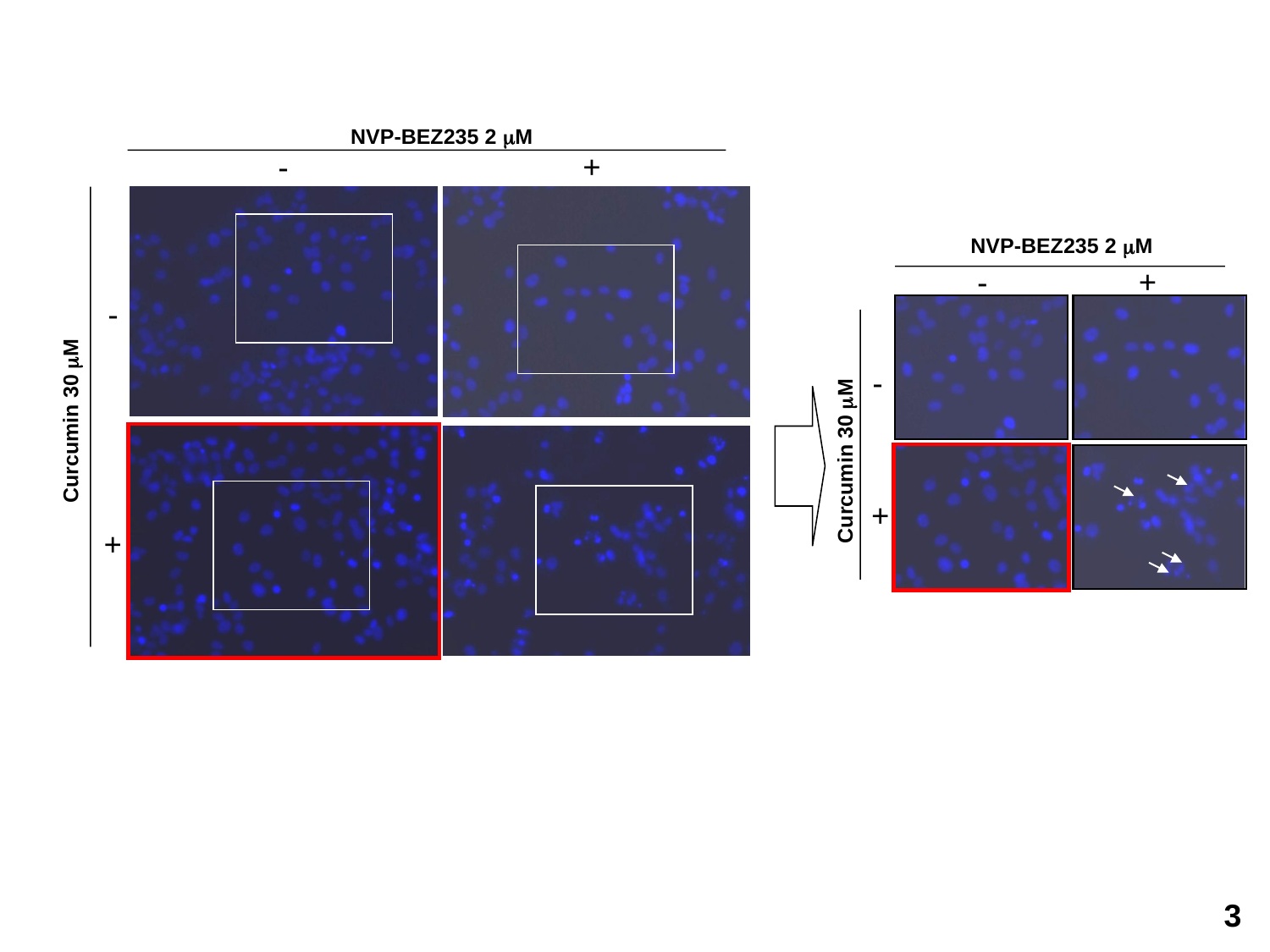

NVP-BEZ235 2 μM
-
+
NVP-BEZ235 2 μM
-
+
-
-
Curcumin 30 μM
Curcumin 30 μM
+
+
3

## Slide 4
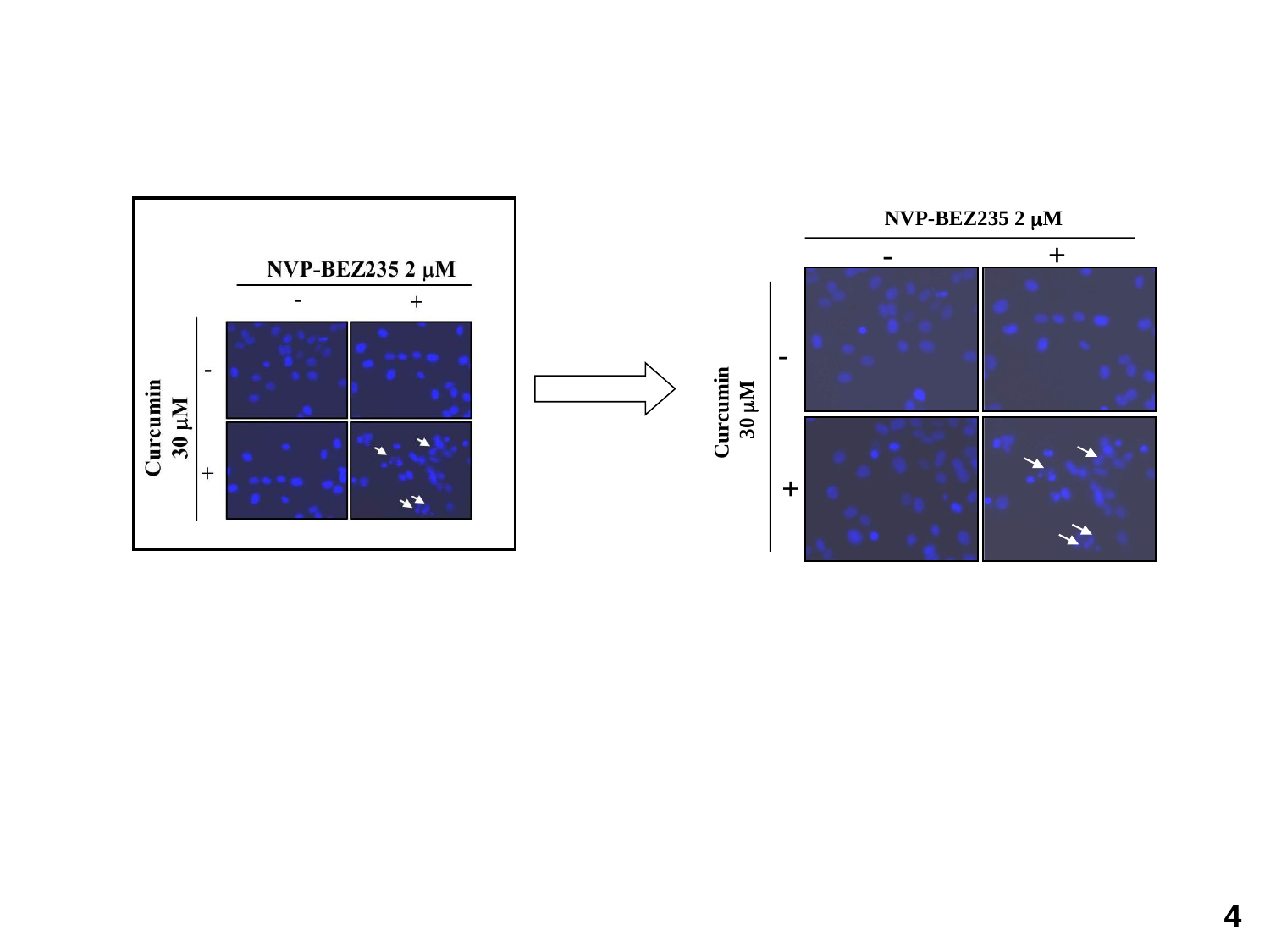

NVP-BEZ235 2 μM
-
+
-
Curcumin
30 μM
+
4
